# Healthcare Service Utilization by Patients with Obstructive Sleep Apnea: A Population-Based Study

**DOI:** 10.1371/journal.pone.0137459

**Published:** 2015-09-04

**Authors:** Li-Ting Kao, Hsin-Chien Lee, Herng-Ching Lin, Ming-Chieh Tsai, Shiu-Dong Chung

**Affiliations:** 1 Graduate Institute of Life Science, National Defense Medical Center, Taipei, Taiwan; 2 Sleep Research Center, Taipei Medical University Hospital, Taipei, Taiwan; 3 Department of Psychiatry, School of Medicine, Taipei Medical University, Taipei, Taiwan; 4 Department of Internal Medicine, Cathay General Hospital, Taipei, Taiwan; 5 School of Medicine, Fu-Jen Catholic University, Hsingchuang, Taiwan; 6 Department of Surgery, Far Eastern Memorial Hospital, Banciao, New Taipei City, Taiwan; University of Rome Tor Vergata, ITALY

## Abstract

**Objective:**

Although obstructive sleep apnea (OSA) is not a life-threatening disease, very few studies have compared differences in healthcare service utilization between patients with and those without OSA in an Asian population according to different age groups. This study attempted to investigate differences in healthcare service utilization between patients with and those without OSA in different age groups in Taiwan.

**Methods:**

Sampled subjects and data on their health service utilization were retrieved from the Taiwan Longitudinal Health Insurance Database 2005. We included 568 patients with OSA and 2840 subjects without OSA. Each subject was followed for a 1-year period to evaluate their healthcare resource utilization. Wilcoxon-Mann-Whitney tests were performed to compare differences in healthcare utilization between patients with and those without OSA during the 1-year follow-up period.

**Results:**

As to all healthcare service utilization, patients with OSA had significantly more outpatient visits (30.3 vs. 18.6), outpatient costs (US$1231.2 vs. US$764.8), inpatient days (1.8 vs. 1.2), inpatient costs (US$563.6 vs. US$276.7), and total costs (US$1794.8 vs. US$1041.5) than comparison subjects during the 1-year follow-up period. Moreover, patients with OSA aged 40~49 and 50~59 years respectively incurred 2.11- and 2.02-fold higher total costs compared to patients without OSA. However, patients with OSA aged over 70 years did not have higher total costs compared to those without OSA.

**Conclusions:**

This study found that patients with OSA had greater healthcare service utilization than those without OSA. Additionally, patients with OSA in the 40~49- and 50~59-year age groups had about 2-fold higher total costs of healthcare services than those without OSA.

## Introduction

Obstructive sleep apnea (OSA) is a prevalent chronic disease which is characterized by recurrent collapse of the upper airway during sleep [1, 2]. It affects approximately 3%~7% of males and 2%~5% of females in adult populations, and usually leads to daytime sleepiness, a low quality of life, lower productivity, and elevated morbidity from cardiovascular diseases (CVDs) and metabolic syndromes [3–9]. Even though OSA is not an immediate life-threatening disease, prior studies found that OSA might contribute to financial burdens on individuals and society [3, 10]. Accordingly, evaluating the substantial economic and social impacts on patients with OSA has become an essential issue.

To date, some studies investigated the influence of OSA on the economy. They consistently found that patients with OSA had higher healthcare service utilization, such as medical costs, medication use, emergency department visits, and hospitalization compared to subjects without OSA in the US, Denmark, Israel, and Canada [11–27]. However, although a variety of studies explored disparities in healthcare service utilization between patients with and those without OSA, all such studies were conducted in Western countries. Additionally, almost no study has estimated differences in healthcare service utilization between patients with and those without OSA in different age groups. Only one study reported that patients aged ≤65 years with OSA used more healthcare services than control subjects [15]; however, in patients over 65 years old, the healthcare costs of patients with OSA were similar to those of control subjects.

The aim of this population-based study was to investigate differences in healthcare service utilization between patients with and those without OSA in Taiwan. We also compared differences in total costs of healthcare services between patients with and those without OSA according to age group. Data from Taiwan present a unique opportunity to explore the association of healthcare service utilization with OSA. Taiwan initiated the National Health Insurance (NHI) program in March 1995 to finance health care for all citizens of Taiwan. Taiwan’s NHI has a unique combination of characteristics including universal coverage, a single-payer payment system with the government as the sole insurer, comprehensive benefits, very low out-of-pocket payment, access to any medical institution of the patient’s choice, and a wide variety of providers well distributed throughout the country. This study should provide some valuable information for health policy decision-makers and medical professionals.

## Methods

### Database

Sampled patients and records of their health service utilization were derived from the Taiwan Longitudinal Health Insurance Database 2005 (LHID2005). The LHID2005 contains longitudinal data on medical claims for 1 million individuals. These 1 million enrollees were randomly selected from the 2005 Registry of Beneficiaries (*n* = 25.68 million) of the Taiwan NHI program. To date, many studies have been published in international peer-reviewed journals employing data from the NHI program [28]. The LHID2005, which was open to the researchers in Taiwan, was available from the Taiwan National Health Research Institute. Only citizens of the Taiwan who fulfill the requirements of conducting research projects are eligible to apply for the National Health Insurance Research Database (http://nhird.nhri.org.tw/en/Data_Protection.html).

The LHID2005 consists of de-identified secondary data released to the public for research purposes, so, after consulting the director of the Institutional Review Board (IRB) of National Defense Medical Center, this study was exempted from full review.

### Study sample

This population-based cross-sectional study included a study group and a comparison group. For the study group, we first selected 1011 patients who were diagnosed with OSA (ICD-9-CM code 327.23, 780.51, 780.53, or 780.57) in ambulatory care visits (including all outpatient visits in hospitals and clinics) between January 1 and December 31, 2011. The date of the first diagnosis of OSA was defined as the index date. In Taiwan, when a physician suspects that a subject has OSA, he or she may give the subject a temporary diagnosis of OSA in order to perform related clinical or lab tests for confirmation. Therefore, we excluded patients who had only one diagnosis of OSA (*n* = 414) during the study period in order to increase the validity of the OSA diagnosis. In other words, this study only included patients who had received OSA diagnoses following polysomnography in order to better ensure the validity of the OSA diagnosis. With the purpose of assuring equal follow-up periods (1 year) for all selected patients, we then excluded patients who died during the year 2012 (*n* = 8). In order to limit the study sample to the adult population, we also ruled out patients aged <18 years (*n* = 21). Ultimately, 568 patients with OSA were included in the study group.

Comparison subjects were also retrieved from the LHID2005. We first identified all subjects who had ambulatory care visits between January 1 and December 31, 2011. We then excluded subjects who had a medical history of OSA since the beginning of the NHI program. We further excluded those subjects who died during the year 2012. We then selected 2840 comparison subjects (five comparison subjects per OSA patient) from the remaining individuals matched with study patients by sex, age group (18~29, 30~39, 40~49, 50~59, 60~69, and ≥70 years), and the month of the index date using the SAS proc surveyselect program (SAS, Cary, NC, USA). We defined the date of their first use of medical services occurring during 2011 as the index date for comparison subjects. Consequently, this study included 3408 study subjects which consisted of 568 patients with OSA and 2840 matched comparison subjects.

### Variables of interest

In this study, all sampled subjects were followed up for a 1-year period starting from the index date. The main variables of healthcare service utilization were defined as follows: numbers of outpatient visits and inpatient days and mean costs of outpatient and inpatient treatments during the 1-year follow-up period. Costs of healthcare services in this study included costs of physician diagnoses, medications, treatments, surgeries, laboratory tests, and diagnostic imaging.

### Statistical analysis

The SAS statistical package (SAS System for Windows, Version 8.2, Cary, NC) was used to perform all analyses in the dataset of this study (S1 File). Chi-squared tests were used to compare differences in patients’ monthly income, geographic location (northern, central, eastern, and southern Taiwan), and urbanization level (5 levels, with 1 being the most urbanized and 5 being the least) between patients with and those without OSA. We then calculated the mean and standard deviation (SD) of all variables of healthcare service utilization. In addition, we performed Wilcoxon-Mann-Whitney tests to investigate differences in variables of healthcare service utilization during the 1-year follow-up period between patients with and those without OSA. Differences were considered significant for two-sided *p* values of <0.05.

## Results

Of the 7298-person study sample, the mean age was 48.4±13.7 years. After matching for sex and age group, there were significant differences in monthly income (*p*<0.001), and geographic region (*p*<0.001) between patients with OSA and comparison subjects (Table 1).

**Table 1:** Demographic characteristics of patients with obstructive sleep apnea (OSA) and comparison subjects (*n* = 3408).

Variable	Patients with OSA (*n* = 568)		Comparison subjects (*n* = 2840)		*p* value
	Total no.	Percent (%)	Total no.	Percent (%)	
Gender					1.000
Male	409	72.0	2045	72.0	
Female	159	28.0	795	28.0	
Age (years)					1.000
18~29	48	8.5	240	8.5	
30~39	102	18.0	510	18.0	
40~49	144	25.4	720	25.4	
50~59	155	27.3	775	27.3	
60~69	86	15.1	430	15.1	
≥70	33	5.8	125	5.8	
Urbanization level					0.139
1 (most urbanized)	193	34.0	890	31.34	
2	170	29.9	822	28.94	
3	98	17.3	480	16.9	
4	67	11.8	351	12.36	
5 (least urbanized)	40	7.0	297	10.46	
Monthly income (US$)					<0.001
$1~530	165	29.1	916	32.3	
$530~830	154	27.1	1001	35.3	
≥$830	249	43.9	923	32.5	
Geographic region					<0.001
Northern	282	49.7	1378	48.5	
Central	192	33.8	649	22.9	
Southern	85	15.0	756	26.6	
Eastern	9	1.6	57	2.0	

Table 2 presents healthcare service utilization in the 1-year period following the index date for patients with and those without OSA. As for all healthcare service utilization, patients with OSA had significantly more outpatient visits (30.3 vs. 18.6, *p*<0.001), outpatient costs (US$1231.2 vs. US$764.8, *p*<0.001), inpatient days (1.8 vs. 1.2, *p*<0.001), inpatient costs (US$563.6 vs. US$276.7, *p*<0.001), and total costs (US$1794.8 vs. US$1041.5, *p*<0.001) than comparison subjects. Total costs for overall healthcare services were about 1.7-fold higher for patients with OSA than comparison subjects. Additionally, after excluding the health services related to the diagnosis of OSA, we still found that patients with OSA had significantly more outpatient visits (29.1 vs. 18.6, p<0.001), outpatient costs (US$1178.6 vs. US$764.6, p<0.001), inpatient days (1.8 vs. 1.2, p<0.001), inpatient costs (US$555.6 vs. US$276.7, p<0.001), and total costs (US$1734.1 vs. US$1041.3, p<0.001) than comparison subjects.

**Table 2:** Use and costs of healthcare services within 1 year by patients with obstructive sleep apnea (OSA) and comparison subjects (*n* = 3408).

Variable	Patients with OSA (*n* = 568)		Comparison subjects (*n* = 2840)		*p* value
	Mean	SD	Mean	SD	
All health services					
Outpatient visits (no.)	30.3	26.1	18.6	17.9	<0.001
Outpatient costs (US$)	1231.2	2495.5	764.8	2340.2	<0.001
Inpatient days (no.)	1.8	11.3	1.2	9.5	<0.001
Inpatient costs (US$)	563.6	5339.0	276.7	1975.6	<0.001
Total costs (US$)	1794.8	6062.8	1,041.5	3306.9	<0.001
All health services (excluding the health services related the diagnosis of OSA)					
Outpatient visits (no.)	29.1	25.5	18.6	17.9	<0.001
Outpatient costs (US$)	1178.6	2482.5	764.6	2340.2	<0.001
Inpatient days (no.)	1.8	11.3	1.2	9.5	<0.001
Inpatient costs (US$)	555.6	5339.0	276.7	1975.6	<0.001
Total costs (US$)	1734.1	6058.6	1041.3	3306.9	<0.001

SD, standard deviation.

Table 3 further displays differences in total costs of healthcare service utilization within the 1-year period between patients with and those without OSA according to different age groups. Patients with OSA aged 40~49 years incurred 2.11-fold higher total costs for overall healthcare services compared to comparison subjects (US$1407.3 vs. US$667.2). We also found that total costs were 2.02-fold greater for patients with OSA than comparison subjects who were 50~59 years old (US$2377.9 vs. US$1176.5). Moreover, total costs were 1.79-, 1.51-, and 1.76-fold higher for patients with OSA than comparison subjects who were 18~29, 30~39, and 60~69 years old (US$646.5 vs. US$361.0; US$666.4 vs. US$440.2; US$3085.7 vs. US$1750.8), respectively. However, patients with OSA aged over 70 years did not have more total costs for overall healthcare services than comparison subjects (US$2540.5 vs. US$3041.0).

**Table 3:** Total costs of healthcare services within 1 year by patients with obstructive sleep apnea (OSA) and comparison subjects according to age group.

Age (years)	Total costs (US$)						Cost ratio^†^
	Patients with OSA (*n* = 568)			Comparison subjects (*n* = 2840)			
	*n*	Mean	SD	*n*	Mean	SD	
18~29	48	646.5	882.1	240	361.0	885.7	1.79
30~39	102	666.4	918.0	510	440.2	1330.4	1.51
40~49	144	1407.3	3302.3	720	667.2	2802.0	2.11
50~59	155	2377.9	10,041.8	755	1176.5	3416.3	2.02
60~69	86	3085.7	6002.8	430	1750.8	3989.0	1.76
≥70	33	2540.5	2088.4	165	3041.0	6610.1	0.84

SD, standard deviation.

^†^Calculated as mean total costs for patients with OSA/comparison subjects.

## Discussion

This population-based study found that patients with OSA had higher healthcare service utilization, including outpatient visits, outpatient costs, inpatient visits, inpatient costs, and total costs compared to patients without OSA. These findings are consistent with those of the previous literature in Western countries. For instance, studies in the US reported that patients with OSA had greater mean medical costs, and more emergency department visits and hospitalizations than those without OSA [11, 12]. Danish studies also showed that patients with OSA had significantly higher rates of health-related contact, medication use, unemployment, etc. before the first OSA diagnosis compared to subjects without OSA [13, 14]. Additionally, many studies in Canada found that patients with OSA had higher physician claims and hospital admissions than those without OSA [19–27]. Furthermore, in Israel, serial studies displayed that healthcare service utilization was heavier in patients with OSA than in subjects without OSA among different populations, such as middle-aged, elderly, male, and female [15–18].

It is not surprising that patients with OSA had greater healthcare service utilization than those without OSA. This might have been caused by elevated incidences of comorbidities in patients with OSA, because repetitive intermittent hypoxia and brief arousal due to OSA can lead to systemic inflammation, oxidative stress, endothelial dysfunction, metabolic dysregulation, etc. [29, 30]. Moreover, these features were suggested to be potential risk factors for the progression of comorbidities. Previous studies consistently reported that patients with OSA had a significantly increasing occurrence of comorbidities, such as CVDs, metabolic syndromes, and mental illness compared to a healthy population [31–34]. In addition, some studies further showed that patients with OSA accompanied by comorbid conditions might carry a significantly greater burden of healthcare service utilization than those without OSA [12, 35].

Additionally, the present study evaluated differences in healthcare services between patients with and those without OSA in different age groups. We found that patients with OSA in the 40~49- and 50~59-year age groups had approximately 2-fold higher total costs of healthcare services than comparison subjects. However, patients aged over 70 years with OSA did not have higher total costs of healthcare services than those without OSA. This finding is comparable to observations by Tarasiuk et al., who found that 2.2-fold more healthcare resources were used by patients ≤65 years old with OSA than control subjects [15]. However, in patients >65 years of age, healthcare costs of patients with OSA and control subjects were similar. Possible explanations might be that differences in comorbidity incidences, cognitive function, and quality of life between patients with and those without OSA are more apparent in middle-aged populations than in elderly populations. One previous study explained how middle-aged patients (40~64 years old) with OSA were likely to be diagnosed with numerous comorbidities, including CVDs, hypertension, hyperlipidemia, diabetes mellitus, asthma, etc. [18]. However, elderly patients (67~89 years old) with OSA only had higher risks of CVDs and hyperlipidemia compared to those without OSA [18]. In addition, a study in the US also reported that disparities in cognitive function and quality of life between patients with high and low risks of OSA were most obvious in the middle-aged group, but the effect was attenuated in those aged over 70 years [36].

The principle strength of this study was the use of a population-based dataset with widespread health benefit coverage and a single-payer system in Taiwan. The characteristics of the LHID2005 database could minimize the potential effect of a selection bias. This database also provided a sufficient sample size and increased the statistical power for evaluating the utilization of healthcare services in patients with and those without OSA. Moreover, the LHID2005 database includes complete utilization information and medical costs for all individuals since they began participating in the NHI program in Taiwan. Thus, the potential risk of a recall bias was entirely avoided in this study. Additionally, most of the residents in our study were ethnic Chinese, and there is a deficit of previous studies of the healthcare service utilization in patients with OSA in Asian countries. There is increasing evidence of phenotypic differences between Caucasian and Chinese patients with OSA according to a recent study [37]. With similar degrees of OSA severity, Chinese patients were identified as having more craniofacial bony restriction, but Caucasian patients were more overweight [37]. Therefore, the results in our study can possibly be generalized to Asian ethnic groups worldwide.

Nevertheless, there are several limitations to our study which need to be considered. First, the LHID2005 database used in this study provides no records of health habits or the body mass index. These are factors that could potentially increase the use of healthcare services in patients with OSA. Second, the LHID2005 which is released by the NHI administration might not contain all patients with OSA. Some patients with mild symptoms of OSA might not seek healthcare services. Thirdly, the income difference between patients with OSA and those without OSA might contribute to the difference in the availability of healthcare utilizations. This may compromise the findings of our study. However, the enrollment rate for Taiwan NHI program was over 99.6% in 2012. The Taiwan NHI program provides affordable (out-of-pocket payment was only about US$3 per outpatient visit), easily accessible, and comprehensive medical service for all its citizens. In addition to this, the Taiwanese government provides premium subsidies for patients with low income. Therefore, the income difference might not bias the findings in this study. Finally, this study did not evaluate indirect costs, such as work-related injuries, lost productivity, or loss of quality of life, in patients with OSA.

In conclusion, this population-based study revealed that patients with OSA had greater healthcare service utilization than those without OSA. Additionally, patients with OSA in the 40~49- and 50~59-year age groups had about 2-fold higher total costs of healthcare services than those without OSA, but patients with OSA over 70 years old did not have higher total costs of healthcare services than comparison subjects. Based on the results of our study, we suggest that policymakers and medical professionals should provide more premium subsidies for relevant treatments for OSA in the middle-aged group, especially for patients 40~49 and 50~59 years old. Physicians are also recommended to provide regular examinations and follow-up for middle-aged patients with OSA. However, further studies are still suggested to investigate the potential factors leading to elevated healthcare service utilization by patients with OSA.

## Supporting Information

S1 File Minimal dataset for this study. (XLS)
